# Supplementary material for: The causal relationship between the human gut microbiota and pyogenic arthritis: a Mendelian randomization study
Source: Front Cell Infect Microbiol. 2024 Nov 26;14:1452480. doi: 10.3389/fcimb.2024.1452480 (PMC11629706; doi:10.3389/fcimb.2024.1452480)
Supplement: Supplementary file 1 [file DataSheet1.zip › mendelian test/ebi-a-GCST90027487.csv_forest.pdf]

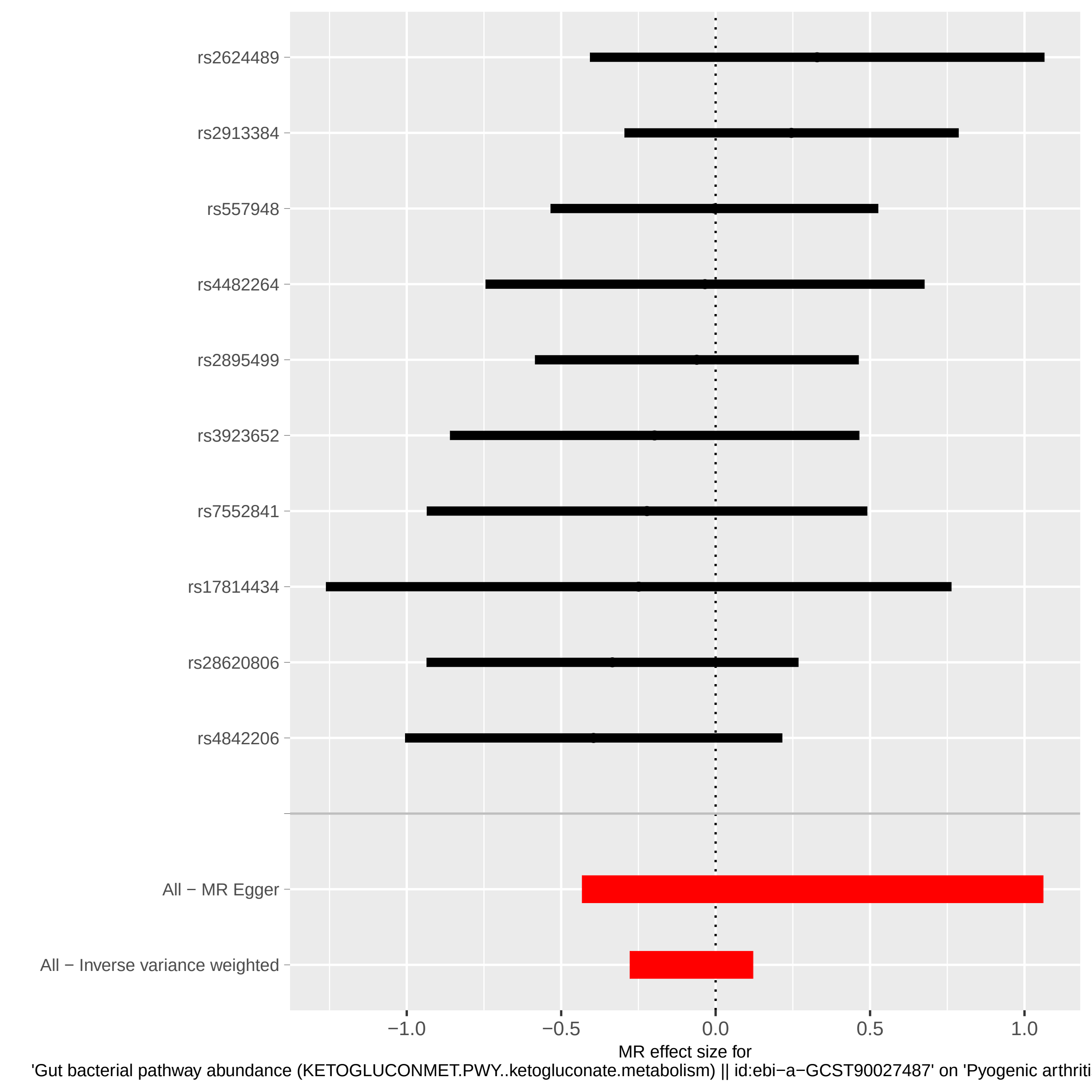
rs2624489
rs2913384
rs557948
rs4482264
rs2895499
rs3923652
rs7552841
rs17814434
rs28620806
rs4842206
All − MR Egger
All − Inverse variance weighted
−1.0
−0.5
0.0
0.5
1.0
MR effect size for
'Gut bacterial pathway abundance (KETOGLUCONMET.PWY..ketogluconate.metabolism) || id:ebi−a−GCST90027487' on 'Pyogenic arthriti
